# Outbreak of leptospirosis amidst COVID’19 in Tanzania: a new threat? Short communication

**DOI:** 10.1097/MS9.0000000000000715

**Published:** 2023-04-25

**Authors:** Omer A. Shaikh, Umama Azam, Muhammad U. Naseem, Sidhant Ochani, Md. Al Hasibuzzaman, Kaleem Ullah

**Affiliations:** a Department of Medicine, Ziauddin University; b Department of Medicine, Karachi Medical and Dental College, Karachi; c Department of Medicine, Khairpur Medical College, Khairpur Mir’s; d Department of Liver Transplantation, Pir Abdul Qadir Shah Institute of Medical Sciences, Gambat, Pakistan; e Institute of Nutrition and Food Science, University of Dhaka, Dhaka, Bangladesh

**Keywords:** Africa, COVID-19, disease X, infectious diseases, eptospirosis, leptospira interrogans, outbreak

## Abstract

As the world is still fighting to combat the coronavirus disease 2019 (COVID-19) virus, the United Republic of Tanzania has been confronting yet another bacterial infection called leptospirosis (LS). It is caused by the spirochete bacteria of genus *Leptospira*, and has been known to infect several people, already claiming a number of lives. It infects 1 million people annually with ~60 000 deaths having a fatality rate of 6.85% worldwide. COVID has profusely burdened the healthcare system worldwide within the past 2 years; it has sabotaged medical management and brought down resources, which has now made it difficult for any country to withstand another pandemic. LS has overburdened the medical care system of Tanzania abjectly; it is now imperative not to overlook environmental factors, like a flood, the presence of rodents, unsatisfactory socioeconomic conditions in areas where dogs reside, substandard wastewater and garbage disposal facilities, or any other factor which might lead to further spread of LS and put Tanzania in jeopardy.

As the world is still fighting to combat the coronavirus disease 2019 (COVID-19) virus, the United Republic of Tanzania (URT) has been confronting yet another bacterial infection called leptospirosis (LS). It is caused by the spirochete bacteria of genus *Leptospira* and has been known to infect several people, already claiming a number of lives. It infects 1 million people annually, with ~60 000 deaths having a fatality rate of 6.85% worldwide^1^. Fears about an outbreak have been raised by a report from the Ministry of Public Health that LS reemerged on 5 July 2022, killing three confirmed cases. The health minister confirmed 20 cases of an outbreak in the southern area of Lindi^2^. More recently, as of 8 August 2022, the URT had documented 20 LS cases, including three deaths. Fifteen of the 20 cases were laboratory-confirmed, and the majority of the cases were men aged 18–77^3^.

The infection causes symptoms including mild ones like nose bleeds, fever, fatigability, and headaches; red eyes, abdominal pain, diarrhea, rash, etc., to severe symptoms, if left untreated, such as pulmonary hemorrhagic syndrome, renal and liver dysfunctions, jaundice, multiple organ dysfunction, or no symptoms at all^4^. The mode of transmission is via animal to human, through either urine of infected animals or other body fluids except for saliva or contact with water, soil, or food contaminated with the urine of diseased animals^5^.

LS in Tanzania was reported in the early 1990s, and now it has been declared as an outbreak by WHO^5^. The main cause of LS infection in Tanzania is a lack of sanitation and the general population’s utilization of contaminated water. Tanzania’s tropical geography, frequent heavy rainfalls, excessive urbanization, wildlife intervention, flooding, and poor management of these aforementioned concerns are the primary drivers of the spirochetes’ exponential rise in Tanzania. As a result of the degradation of natural habitats in Tanzania, wildlife has been compelled to dwell closer to human settlements, raising the probability of *Leptospira interrogans* infection in the general population^6^.

The season from May to Oct is a dry season with cooler temperatures in Tanzania, and as this disease is known to be a greater risk in the warmer season, this is high time to take active measures to prevent the further spread of this disease, by carrying out most awareness programs, guiding people about the proper sanitary measures that should be taken to avoid contact with the bacteria since the source of the outbreak remains unidentified. More importantly, farmers, mine workers, sewer workers, slaughterhouse workers, veterinarians and animal caretakers, fish workers, and cattlemen must be made well aware and mindful as they are most exposed to animals and other sources of spread of this infection like contaminated soil and water^4^. Therefore, it should be made sure that they are provided with protective clothing or footwear to lessen the exposure to a greater extent.

COVID has profusely burdened the healthcare system worldwide within the past 2 years; it has sabotaged medical management and brought down resources, which has now made it difficult for any country to withstand another pandemic. LS has overburdened the medical care system of Tanzania abjectly; it is now imperative not to overlook environmental factors, like flood, presence of rodents, unsatisfactory socioeconomic conditions in areas where dogs reside, substandard wastewater and garbage disposal facilities, or any other factor which might lead to further spread of LS, and put Tanzania in jeopardy. Any information regarding risk factors of LS in these times will help the head of health services to take necessary preventive and prophylactic measures in the spread of this disease in the state. This disease has a greater risk of spreading in urban and peri-urban areas; the incidence of LS is increasing in children living in these areas. People of Tanzania should be made aware that this disease is also associated with swimming, rafting, drifting, rowing, or wading in contaminated rivers and lakes; therefore, they should take the required safety measures and help in limiting its spread. Moreover, looking at the previous outbreaks, Africa being the hub of infectious diseases, needs special attention, adequate resources, and a reformed healthcare system without inequity, as was seen during COVID vaccine rollout. Therefore WHO, along with other countries, should help Africa in aspects of its development and prevention of the next outbreak, of Disease X.

## Ethical approval

Not applicable.

## Consent

Not applicable.

## Sources of funding

The author(s) received no financial support for the research, authorship, and/or publication of this article.

## Author contribution

O.H.S., U.A., M.U.N. and S.O.: writing of the manuscript; S.O., M.A.H., and K.U.: review editing, formatting, and referencing.

## Conflicts of interest disclosure

The author(s) declared no potential conflicts of interest concerning the research, authorship, and/or publication of this article.

## Research registration unique identifying number (UIN)

Not applicable.

## Guarantor

All authors accept full responsibility for the work and/or the conduct of the study, had access to the data, and controlled the decision to publish.

## Provenance and peer review

Not commissioned, externally peer-reviewed.

## Data availability statement

Available upon reasonable request from the corresponding author.
